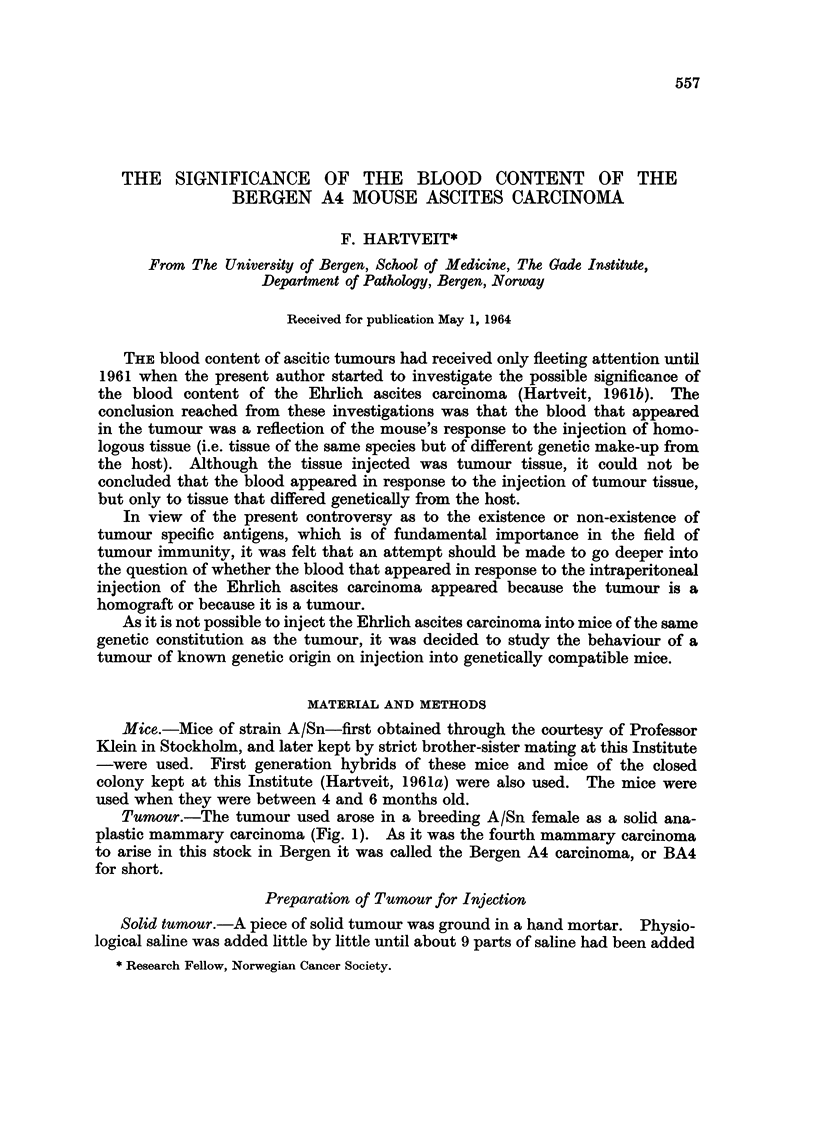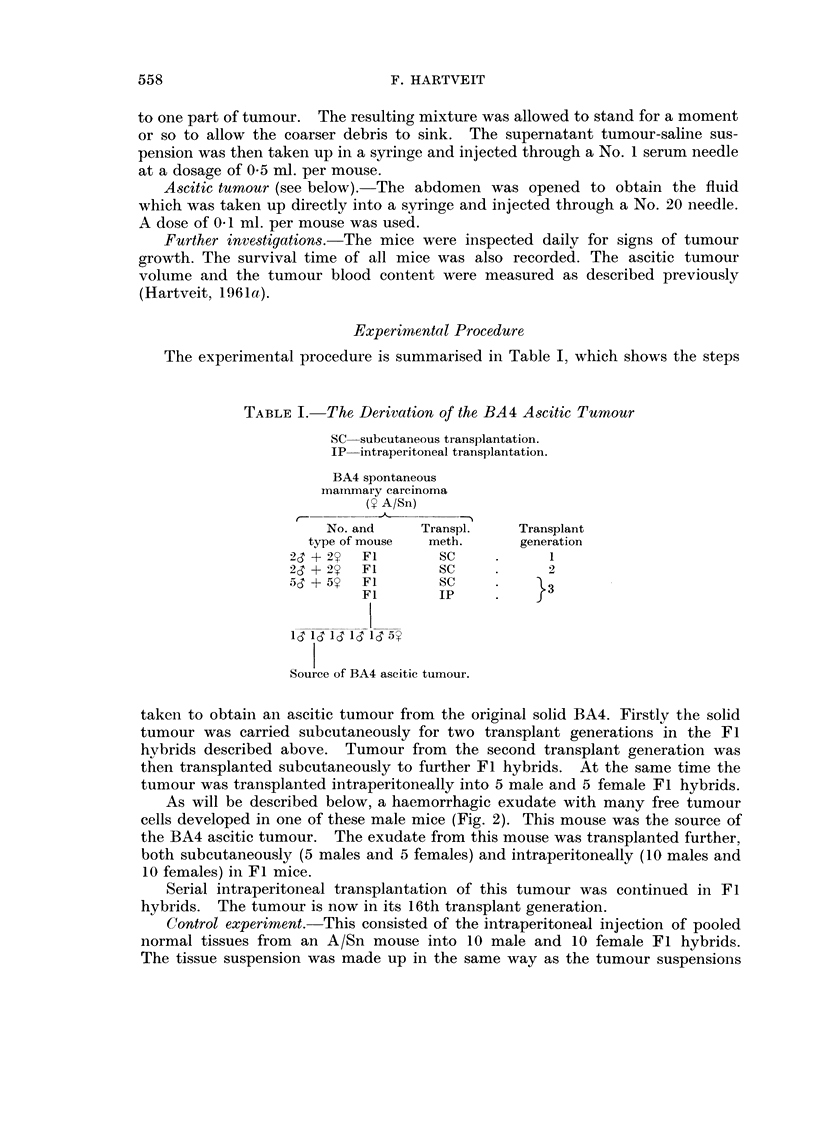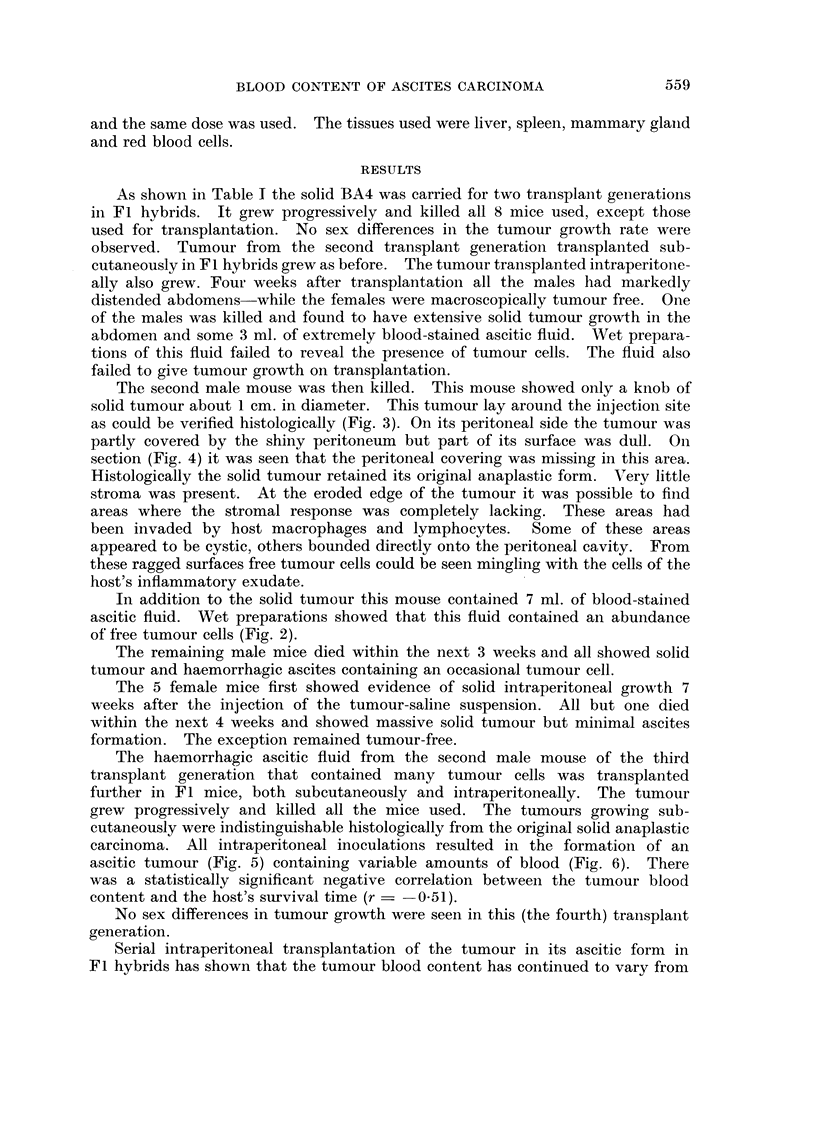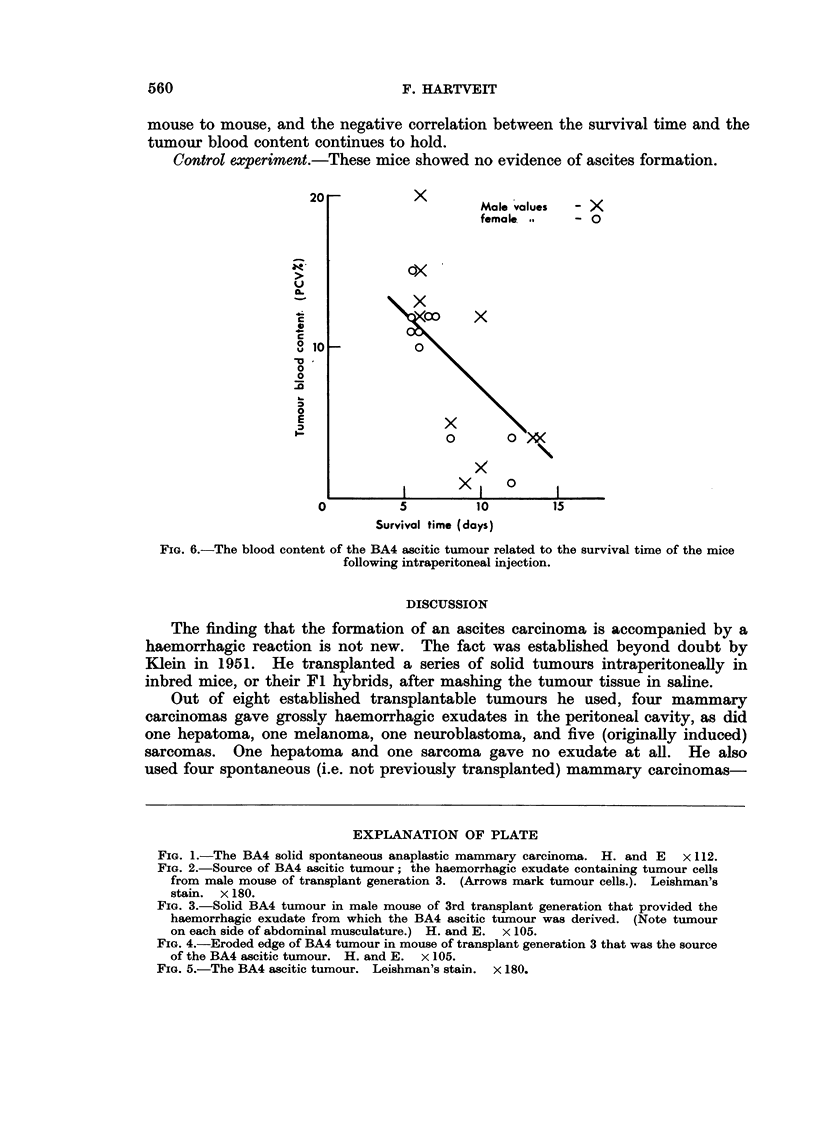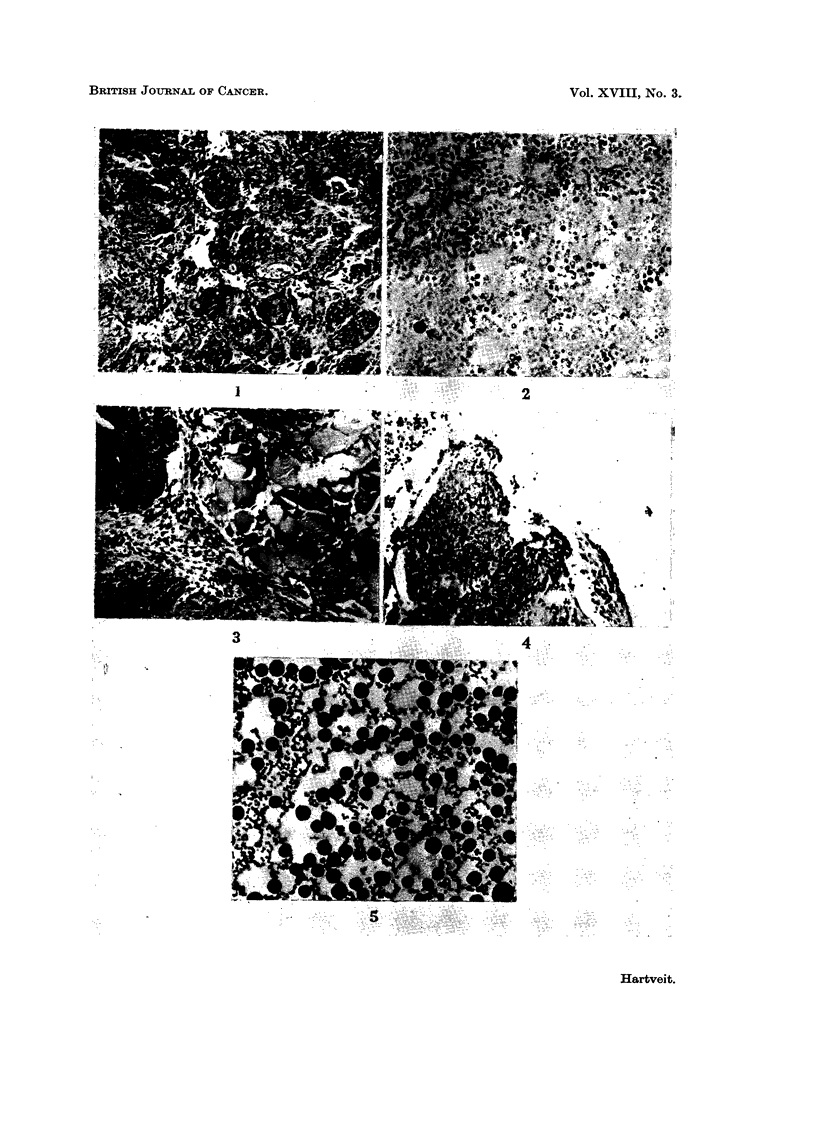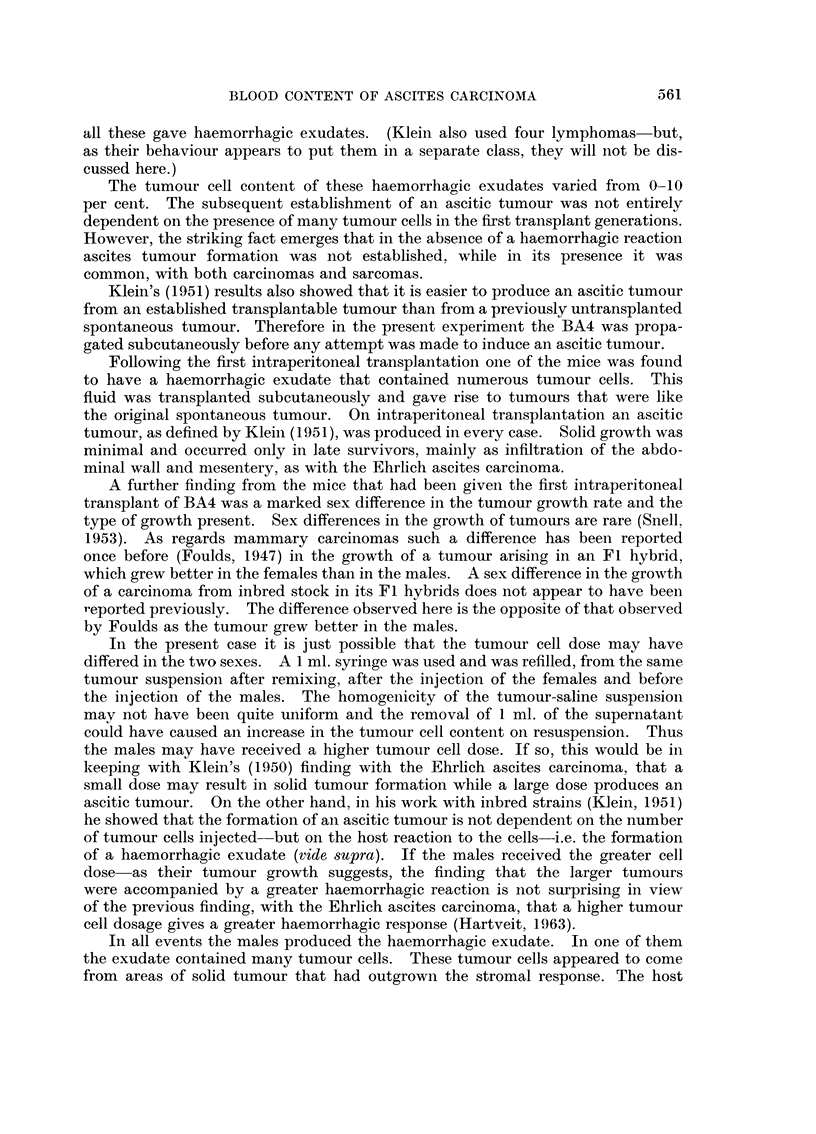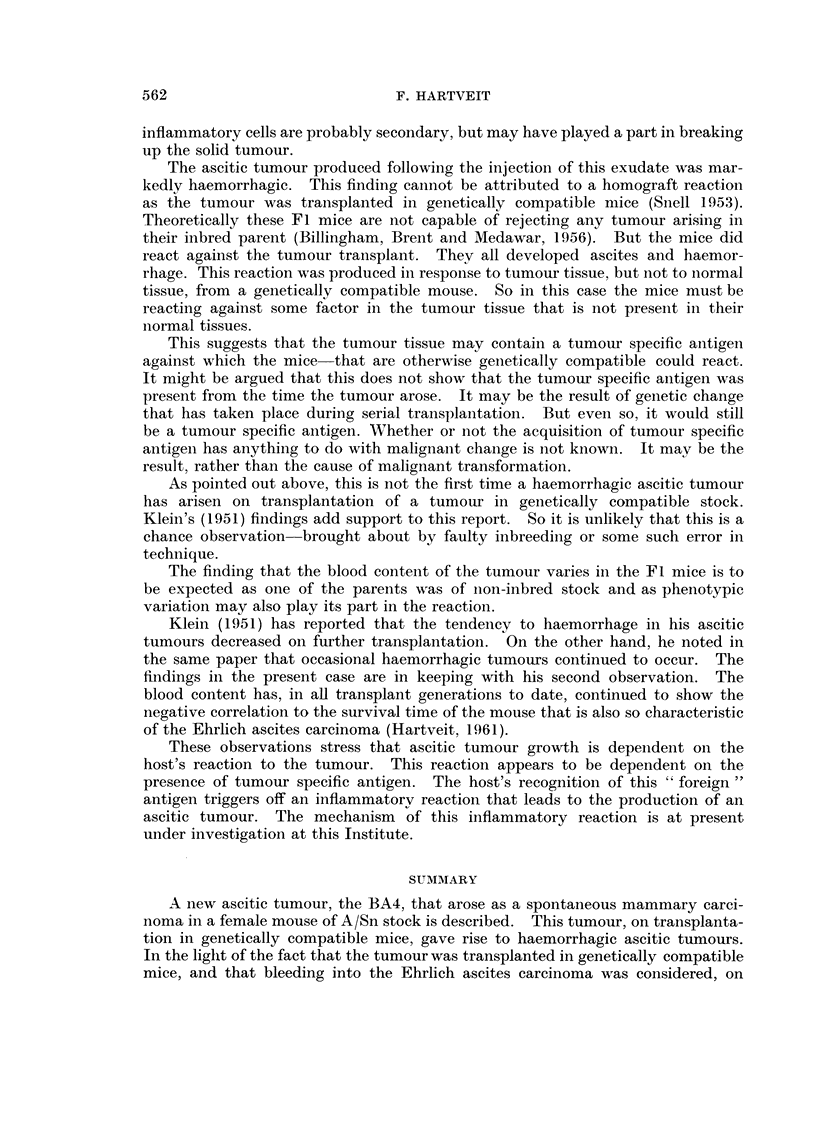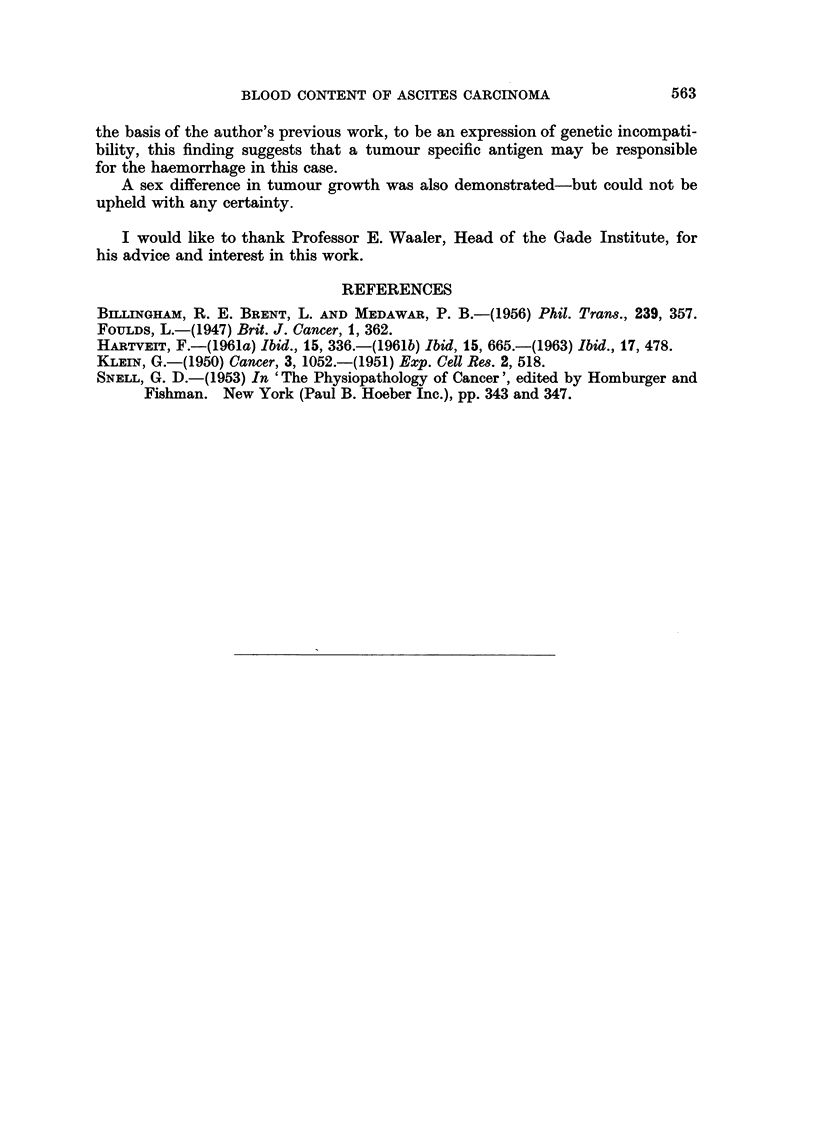# The Significance of the Blood Content of the Bergen A4 Mouse Ascites Carcinoma

**DOI:** 10.1038/bjc.1964.64

**Published:** 1964-09

**Authors:** F. Hartveit

## Abstract

**Images:**


### THE SIGNIFICANCE OF THE BLOOD CONTENT OF THE BERGEN A4 MOUSE ASCITES CARCINOMA

F. HARTVEIT*

*From The University of Bergen, School of Medicine, The Gade Institute, Department of Pathology, Bergen, Norway*

Received for publication May 1, 1964

THE blood content of ascitic tumours had received only fleeting attention until 1961 when the present author started to investigate the possible significance of the blood content of the Ehrlich ascites carcinoma (Hartveit, 1961*b*). The conclusion reached from these investigations was that the blood that appeared in the tumour was a reflection of the mouse's response to the injection of homologous tissue (i.e. tissue of the same species but of different genetic make-up from the host). Although the tissue injected was tumour tissue, it could not be concluded that the blood appeared in response to the injection of tumour tissue, but only to tissue that differed genetically from the host.

In view of the present controversy as to the existence or non-existence of tumour specific antigens, which is of fundamental importance in the field of tumour immunity, it was felt that an attempt should be made to go deeper into the question of whether the blood that appeared in response to the intraperitoneal injection of the Ehrlich ascites carcinoma appeared because the tumour is a homograft or because it is a tumour.

As it is not possible to inject the Ehrlich ascites carcinoma into mice of the same genetic constitution as the tumour, it was decided to study the behaviour of a tumour of known genetic origin on injection into genetically compatible mice.

### MATERIAL AND METHODS

*Mice.*—Mice of strain A/Sn—first obtained through the courtesy of Professor Klein in Stockholm, and later kept by strict brother-sister mating at this Institute —were used. First generation hybrids of these mice and mice of the closed colony kept at this Institute (Hartveit, 1961*a*) were also used. The mice were used when they were between 4 and 6 months old.

*Tumour.*—The tumour used arose in a breeding A/Sn female as a solid anaplastic mammary carcinoma (Fig. 1). As it was the fourth mammary carcinoma to arise in this stock in Bergen it was called the Bergen A4 carcinoma, or BA4 for short.

### *Preparation of Tumour for Injection*

*Solid tumour.*—A piece of solid tumour was ground in a hand mortar. Physiological saline was added little by little until about 9 parts of saline had been added

* Research Fellow, Norwegian Cancer Society.

to one part of tumour. The resulting mixture was allowed to stand for a moment or so to allow the coarser debris to sink. The supernatant tumour-saline suspension was then taken up in a syringe and injected through a No. 1 serum needle at a dosage of 0·5 ml. per mouse.

*Ascitic tumour* (see below).—The abdomen was opened to obtain the fluid which was taken up directly into a syringe and injected through a No. 20 needle. A dose of 0·1 ml. per mouse was used.

*Further investigations.*—The mice were inspected daily for signs of tumour growth. The survival time of all mice was also recorded. The ascitic tumour volume and the tumour blood content were measured as described previously (Hartveit, 1961*a*).

### *Experimental Procedure*

The experimental procedure is summarised in Table I, which shows the steps

TABLE I.—*The Derivation of the BA4 Ascitic Tumour*

SC—subcutaneous transplantation.
IP—intraperitoneal transplantation.

BA4 spontaneous mammary carcinoma (♀ A/Sn)

| No. and type of mouse | Transpl. meth. | Transplant generation |
|---|---|---|
| 2♂ + 2♀ F1 | SC | 1 |
| 2♂ + 2♀ F1 | SC | 2 |
| 5♂ + 5♀ F1 | SC | } 3 |
| F1 | IP | |

1♂ 1♂ 1♂ 1♂ 1♂ 5♀

Source of BA4 ascitic tumour.

taken to obtain an ascitic tumour from the original solid BA4. Firstly the solid tumour was carried subcutaneously for two transplant generations in the F1 hybrids described above. Tumour from the second transplant generation was then transplanted subcutaneously to further F1 hybrids. At the same time the tumour was transplanted intraperitoneally into 5 male and 5 female F1 hybrids.

As will be described below, a haemorrhagic exudate with many free tumour cells developed in one of these male mice (Fig. 2). This mouse was the source of the BA4 ascitic tumour. The exudate from this mouse was transplanted further, both subcutaneously (5 males and 5 females) and intraperitoneally (10 males and 10 females) in F1 mice.

Serial intraperitoneal transplantation of this tumour was continued in F1 hybrids. The tumour is now in its 16th transplant generation.

*Control experiment.*—This consisted of the intraperitoneal injection of pooled normal tissues from an A/Sn mouse into 10 male and 10 female F1 hybrids. The tissue suspension was made up in the same way as the tumour suspensions and the same dose was used. The tissues used were liver, spleen, mammary gland and red blood cells.

### RESULTS

As shown in Table I the solid BA4 was carried for two transplant generations in F1 hybrids. It grew progressively and killed all 8 mice used, except those used for transplantation. No sex differences in the tumour growth rate were observed. Tumour from the second transplant generation transplanted subcutaneously in F1 hybrids grew as before. The tumour transplanted intraperitoneally also grew. Four weeks after transplantation all the males had markedly distended abdomens—while the females were macroscopically tumour free. One of the males was killed and found to have extensive solid tumour growth in the abdomen and some 3 ml. of extremely blood-stained ascitic fluid. Wet preparations of this fluid failed to reveal the presence of tumour cells. The fluid also failed to give tumour growth on transplantation.

The second male mouse was then killed. This mouse showed only a knob of solid tumour about 1 cm. in diameter. This tumour lay around the injection site as could be verified histologically (Fig. 3). On its peritoneal side the tumour was partly covered by the shiny peritoneum but part of its surface was dull. On section (Fig. 4) it was seen that the peritoneal covering was missing in this area. Histologically the solid tumour retained its original anaplastic form. Very little stroma was present. At the eroded edge of the tumour it was possible to find areas where the stromal response was completely lacking. These areas had been invaded by host macrophages and lymphocytes. Some of these areas appeared to be cystic, others bounded directly onto the peritoneal cavity. From these ragged surfaces free tumour cells could be seen mingling with the cells of the host's inflammatory exudate.

In addition to the solid tumour this mouse contained 7 ml. of blood-stained ascitic fluid. Wet preparations showed that this fluid contained an abundance of free tumour cells (Fig. 2).

The remaining male mice died within the next 3 weeks and all showed solid tumour and haemorrhagic ascites containing an occasional tumour cell.

The 5 female mice first showed evidence of solid intraperitoneal growth 7 weeks after the injection of the tumour-saline suspension. All but one died within the next 4 weeks and showed massive solid tumour but minimal ascites formation. The exception remained tumour-free.

The haemorrhagic ascitic fluid from the second male mouse of the third transplant generation that contained many tumour cells was transplanted further in F1 mice, both subcutaneously and intraperitoneally. The tumour grew progressively and killed all the mice used. The tumours growing subcutaneously were indistinguishable histologically from the original solid anaplastic carcinoma. All intraperitoneal inoculations resulted in the formation of an ascitic tumour (Fig. 5) containing variable amounts of blood (Fig. 6). There was a statistically significant negative correlation between the tumour blood content and the host's survival time ($r = -0{\cdot}51$).

No sex differences in tumour growth were seen in this (the fourth) transplant generation.

Serial intraperitoneal transplantation of the tumour in its ascitic form in F1 hybrids has shown that the tumour blood content has continued to vary from mouse to mouse, and the negative correlation between the survival time and the tumour blood content continues to hold.

*Control experiment.*—These mice showed no evidence of ascites formation.

FIG. 6.—The blood content of the BA4 ascitic tumour related to the survival time of the mice following intraperitoneal injection.

### DISCUSSION

The finding that the formation of an ascites carcinoma is accompanied by a haemorrhagic reaction is not new. The fact was established beyond doubt by Klein in 1951. He transplanted a series of solid tumours intraperitoneally in inbred mice, or their F1 hybrids, after mashing the tumour tissue in saline.

Out of eight established transplantable tumours he used, four mammary carcinomas gave grossly haemorrhagic exudates in the peritoneal cavity, as did one hepatoma, one melanoma, one neuroblastoma, and five (originally induced) sarcomas. One hepatoma and one sarcoma gave no exudate at all. He also used four spontaneous (i.e. not previously transplanted) mammary carcinomas—

---

### EXPLANATION OF PLATE

FIG. 1.—The BA4 solid spontaneous anaplastic mammary carcinoma. H. and E ×112.

FIG. 2.—Source of BA4 ascitic tumour; the haemorrhagic exudate containing tumour cells from male mouse of transplant generation 3. (Arrows mark tumour cells.). Leishman's stain. ×180.

FIG. 3.—Solid BA4 tumour in male mouse of 3rd transplant generation that provided the haemorrhagic exudate from which the BA4 ascitic tumour was derived. (Note tumour on each side of abdominal musculature.) H. and E. ×105.

FIG. 4.—Eroded edge of BA4 tumour in mouse of transplant generation 3 that was the source of the BA4 ascitic tumour. H. and E. ×105.

FIG. 5.—The BA4 ascitic tumour. Leishman's stain. ×180.

1

2

3

4

5

all these gave haemorrhagic exudates. (Klein also used four lymphomas—but, as their behaviour appears to put them in a separate class, they will not be discussed here.)

The tumour cell content of these haemorrhagic exudates varied from 0–10 per cent. The subsequent establishment of an ascitic tumour was not entirely dependent on the presence of many tumour cells in the first transplant generations. However, the striking fact emerges that in the absence of a haemorrhagic reaction ascites tumour formation was not established, while in its presence it was common, with both carcinomas and sarcomas.

Klein's (1951) results also showed that it is easier to produce an ascitic tumour from an established transplantable tumour than from a previously untransplanted spontaneous tumour. Therefore in the present experiment the BA4 was propagated subcutaneously before any attempt was made to induce an ascitic tumour.

Following the first intraperitoneal transplantation one of the mice was found to have a haemorrhagic exudate that contained numerous tumour cells. This fluid was transplanted subcutaneously and gave rise to tumours that were like the original spontaneous tumour. On intraperitoneal transplantation an ascitic tumour, as defined by Klein (1951), was produced in every case. Solid growth was minimal and occurred only in late survivors, mainly as infiltration of the abdominal wall and mesentery, as with the Ehrlich ascites carcinoma.

A further finding from the mice that had been given the first intraperitoneal transplant of BA4 was a marked sex difference in the tumour growth rate and the type of growth present. Sex differences in the growth of tumours are rare (Snell, 1953). As regards mammary carcinomas such a difference has been reported once before (Foulds, 1947) in the growth of a tumour arising in an F1 hybrid, which grew better in the females than in the males. A sex difference in the growth of a carcinoma from inbred stock in its F1 hybrids does not appear to have been reported previously. The difference observed here is the opposite of that observed by Foulds as the tumour grew better in the males.

In the present case it is just possible that the tumour cell dose may have differed in the two sexes. A 1 ml. syringe was used and was refilled, from the same tumour suspension after remixing, after the injection of the females and before the injection of the males. The homogenicity of the tumour-saline suspension may not have been quite uniform and the removal of 1 ml. of the supernatant could have caused an increase in the tumour cell content on resuspension. Thus the males may have received a higher tumour cell dose. If so, this would be in keeping with Klein's (1950) finding with the Ehrlich ascites carcinoma, that a small dose may result in solid tumour formation while a large dose produces an ascitic tumour. On the other hand, in his work with inbred strains (Klein, 1951) he showed that the formation of an ascitic tumour is not dependent on the number of tumour cells injected—but on the host reaction to the cells—i.e. the formation of a haemorrhagic exudate (*vide supra*). If the males received the greater cell dose—as their tumour growth suggests, the finding that the larger tumours were accompanied by a greater haemorrhagic reaction is not surprising in view of the previous finding, with the Ehrlich ascites carcinoma, that a higher tumour cell dosage gives a greater haemorrhagic response (Hartveit, 1963).

In all events the males produced the haemorrhagic exudate. In one of them the exudate contained many tumour cells. These tumour cells appeared to come from areas of solid tumour that had outgrown the stromal response. The host inflammatory cells are probably secondary, but may have played a part in breaking up the solid tumour.

The ascitic tumour produced following the injection of this exudate was markedly haemorrhagic. This finding cannot be attributed to a homograft reaction as the tumour was transplanted in genetically compatible mice (Snell 1953). Theoretically these F1 mice are not capable of rejecting any tumour arising in their inbred parent (Billingham, Brent and Medawar, 1956). But the mice did react against the tumour transplant. They all developed ascites and haemorrhage. This reaction was produced in response to tumour tissue, but not to normal tissue, from a genetically compatible mouse. So in this case the mice must be reacting against some factor in the tumour tissue that is not present in their normal tissues.

This suggests that the tumour tissue may contain a tumour specific antigen against which the mice—that are otherwise genetically compatible could react. It might be argued that this does not show that the tumour specific antigen was present from the time the tumour arose. It may be the result of genetic change that has taken place during serial transplantation. But even so, it would still be a tumour specific antigen. Whether or not the acquisition of tumour specific antigen has anything to do with malignant change is not known. It may be the result, rather than the cause of malignant transformation.

As pointed out above, this is not the first time a haemorrhagic ascitic tumour has arisen on transplantation of a tumour in genetically compatible stock. Klein's (1951) findings add support to this report. So it is unlikely that this is a chance observation—brought about by faulty inbreeding or some such error in technique.

The finding that the blood content of the tumour varies in the F1 mice is to be expected as one of the parents was of non-inbred stock and as phenotypic variation may also play its part in the reaction.

Klein (1951) has reported that the tendency to haemorrhage in his ascitic tumours decreased on further transplantation. On the other hand, he noted in the same paper that occasional haemorrhagic tumours continued to occur. The findings in the present case are in keeping with his second observation. The blood content has, in all transplant generations to date, continued to show the negative correlation to the survival time of the mouse that is also so characteristic of the Ehrlich ascites carcinoma (Hartveit, 1961).

These observations stress that ascitic tumour growth is dependent on the host's reaction to the tumour. This reaction appears to be dependent on the presence of tumour specific antigen. The host's recognition of this " foreign " antigen triggers off an inflammatory reaction that leads to the production of an ascitic tumour. The mechanism of this inflammatory reaction is at present under investigation at this Institute.

### SUMMARY

A new ascitic tumour, the BA4, that arose as a spontaneous mammary carcinoma in a female mouse of A/Sn stock is described. This tumour, on transplantation in genetically compatible mice, gave rise to haemorrhagic ascitic tumours. In the light of the fact that the tumour was transplanted in genetically compatible mice, and that bleeding into the Ehrlich ascites carcinoma was considered, on the basis of the author's previous work, to be an expression of genetic incompatibility, this finding suggests that a tumour specific antigen may be responsible for the haemorrhage in this case.

A sex difference in tumour growth was also demonstrated—but could not be upheld with any certainty.

I would like to thank Professor E. Waaler, Head of the Gade Institute, for his advice and interest in this work.

### REFERENCES

BILLINGHAM, R. E. BRENT, L. AND MEDAWAR, P. B.—(1956) *Phil. Trans.*, **239**, 357.
FOULDS, L.—(1947) *Brit. J. Cancer*, **1**, 362.
HARTVEIT, F.—(1961*a*) *Ibid.*, **15**, 336.—(1961*b*) *Ibid*, **15**, 665.—(1963) *Ibid.*, **17**, 478.
KLEIN, G.—(1950) *Cancer*, **3**, 1052.—(1951) *Exp. Cell Res.* **2**, 518.
SNELL, G. D.—(1953) *In* 'The Physiopathology of Cancer', edited by Homburger and Fishman. New York (Paul B. Hoeber Inc.), pp. 343 and 347.